# (*E*)-1-(4-Chloro­phen­yl)ethanone semi­carbazone

**DOI:** 10.1107/S160053680902279X

**Published:** 2009-06-20

**Authors:** Hoong-Kun Fun, Ching Kheng Quah, Mahesh Padaki, Shridhar Malladi, Arun M. Isloor

**Affiliations:** a X-ray Crystallography Unit, School of Physics, Universiti Sains Malaysia, 11800 USM, Penang, Malaysia; b Department of Chemistry, National Institute of Technology–Karnataka, Surathkal, Mangalore 575 025, India

## Abstract

In the title compound, C_9_H_10_ClN_3_O, the semicarbazone group is approximately planar, with an r.m.s. deviation from the mean plane of 0.054 (1) Å. The dihedral angle between the least-squares planes through the semicarbazone group and the benzene ring is 30.46 (5)°. In the solid state, mol­ecules are linked *via* inter­molecular N—H⋯O and N—H⋯N hydrogen bonds, generating *R*_2_^2^(9) ring motifs which, together with *R*_2_^2^(8) ring motifs formed by pairs of inter­molecular N—H⋯O hydrogen bonds, lead to the formation of a seldom-observed mol­ecular trimer. Furthermore, N—H⋯O hydrogen bonds form *R*_2_^1^(7) ring motifs with C—H⋯O hydrogen bonds, further consolidating the crystal structure. Mol­ecules are linked by these inter­molecular inter­actions, forming two-dimensional networks parallel to (100).

## Related literature

For the synthetic utility and applications of semicarbazone derivatives, see: Warren *et al.* (1977 ▶); Chandra & Gupta (2005 ▶); Jain *et al.* (2002 ▶); Pilgram (1978 ▶); Yogeeswari *et al.* (2004 ▶). For a related structure, see: Fun *et al.* (2009 ▶). For the preparation, see: Furniss *et al.* (1978 ▶). For hydrogen-bond motifs, see: Bernstein *et al.* (1995 ▶). For bond-length data, see: Allen *et al.* (1987 ▶). For the stability of the temperature controller used for the data collection, see: Cosier & Glazer (1986 ▶).
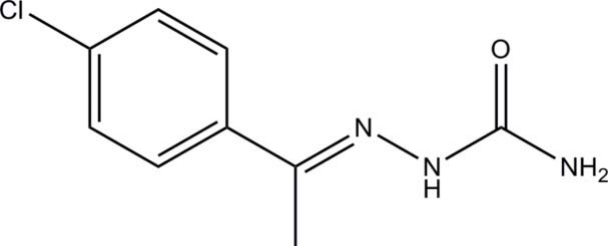


## Experimental

### 

#### Crystal data

C_9_H_10_ClN_3_O
*M* *_r_* = 211.65
Monoclinic, 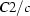

*a* = 21.8191 (4) Å
*b* = 7.0484 (1) Å
*c* = 13.7249 (2) Å
β = 109.633 (1)°
*V* = 1988.04 (6) Å^3^
*Z* = 8
Mo *K*α radiation
μ = 0.35 mm^−1^
*T* = 100 K
0.41 × 0.20 × 0.03 mm

#### Data collection

Bruker SMART APEXII CCD area-detector diffractometer
Absorption correction: multi-scan (**SADABS**; Bruker, 2005 ▶) *T*_min_ = 0.867, *T*_max_ = 0.991
28636 measured reflections
3539 independent reflections
2912 reflections with *I* > 2σ(*I*)
*R*_int_ = 0.052

#### Refinement

*R*[*F* ^2^ > 2σ(*F* ^2^)] = 0.039
*wR*(*F* ^2^) = 0.101
*S* = 1.04
3539 reflections
167 parameters
H atoms treated by a mixture of independent and constrained refinement
Δρ_max_ = 0.45 e Å^−3^
Δρ_min_ = −0.27 e Å^−3^

### 

Data collection: *APEX2* (Bruker, 2005 ▶); cell refinement: *SAINT* (Bruker, 2005 ▶); data reduction: *SAINT*; program(s) used to solve structure: *SHELXTL* (Sheldrick, 2008 ▶); program(s) used to refine structure: *SHELXTL*; molecular graphics: *SHELXTL*; software used to prepare material for publication: *SHELXTL* and *PLATON* (Spek, 2009 ▶).

## Supplementary Material

Crystal structure: contains datablocks global, I. DOI: 10.1107/S160053680902279X/sj2631sup1.cif

Structure factors: contains datablocks I. DOI: 10.1107/S160053680902279X/sj2631Isup2.hkl

Additional supplementary materials:  crystallographic information; 3D view; checkCIF report

## Figures and Tables

**Table 1:** Hydrogen-bond geometry (Å, °)

*D*—H⋯*A*	*D*—H	H⋯*A*	*D*⋯*A*	*D*—H⋯*A*
N2—H1*N*2⋯O1^i^	0.884 (19)	2.007 (19)	2.8866 (12)	173.3 (19)
N3—H1*N*3⋯N1^ii^	0.835 (18)	2.264 (18)	3.0904 (14)	170.5 (16)
N3—H2*N*3⋯O1^iii^	0.826 (17)	2.316 (17)	3.0499 (13)	148.4 (15)
C9—H9*C*⋯O1^i^	0.94 (2)	2.55 (2)	3.2162 (16)	128.1 (16)

Symmetry codes: (i) 
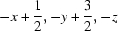
; (ii) 
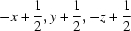
; (iii) 
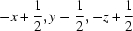
.

**Fig. 1.**
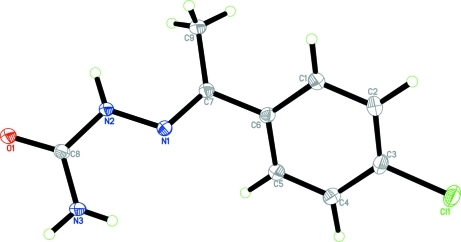
The molecular structure of the title compound, showing 50% probability displacement ellipsoids and the atom-numbering scheme.

**Fig. 2.**
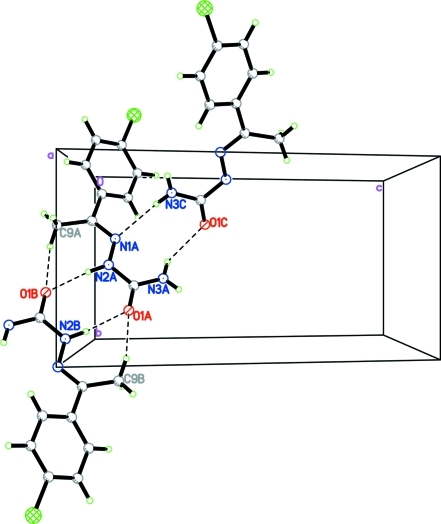
Part of the crystal packing of the title compound, viewed along the a axis, showing the formation of a molecular trimer. Atom-numbering is shown for those non-H atoms involved in hydrogen bonds and intermolecular interactions are shown as dashed lines. Molecule A is is related to molecules B and C via symmetry codes of -x + 1/2, -y + 1/2, -z and -x + 1/2, y + 1/2, -z + 1/2, respectively.

## supplementary crystallographic information

### Comment

In organic chemistry, a semicarbazone is a derivative of an aldehyde or ketone formed by a condensation between a ketone or aldehyde and semicarbazide. Semicarbazones find numerous applications in the field of synthetic chemistry, such as medicinal chemistry (Warren *et al.*, 1977), organometalics (Chandra & Gupta, 2005), polymers (Jain *et al.*, 2002) and herbicides (Pilgram, 1978). 4-Sulphamoylphenyl semicarbazones were synthesized and were found to possess anticonvulsant activity (Yogeeswari *et al.*, 2004). Herein we report the crystal structure of the title semicarbazone which may have commercial and synthetic importance.

The bond lengths (Allen *et al.*, 1987) and angles in the molecule (Fig. 1) are within normal ranges, and are comparable to those observed in a closely related structure (Fun *et al.*, 2009). The semicarbazone group (C9/C6/C7/N1/N2/C8/O1/N3) is approximately planar, with an r.m.s. deviation of 0.054 (1) Å for atom N2 while the dihedral angle between the least-squares planes through the semicarbazone group and the benzene ring is 30.46 (5)°.

In the solid state, the molecules are linked *via* intermolecular N3—H2N3···O1 and N3—H1N3···N1 hydrogen bonds to generate *R*_2_^2^(9) ring motifs which, together with the *R*_2_^2^(8) ring motifs formed by pairs of intermolecular N2—H1N2···O1 hydrogen bonds, lead to the formation of a seldom-observed molecular trimer (Fig. 2). Furthermore, N2—H1N2···O1 hydrogen bonds form *R*_2_^1^(7) ring motifs (Fig. 2) with C9—H9C···O1 hydrogen bonds to further consolidated the crystal structure. The molecules are linked by these intermolecular interactions to form two-dimensional networks parallel to the (1 0 0) plane.

### Experimental

0.780 g (7.0 mmol) of semicarbazide hydrochloride and 0.698 g (8.5 mmol) of crystallized sodium acetate was dissolved in 10 ml of water (Furniss *et al.*, 1978). The reaction mixture was stirred at room temperature for 10 minutes. To this (1 g, 6.5 mmol) of 4-choloacetophenone was added and shaken well. A little alcohol was added to dissolve the turbidity. It was shaken for 10 more minutes and allowed to stand. The semicarbazone crystallizes on standing for 6 h. The separated crystals were filtered, washed with cold water and recrystallized from alcohol. Yield was found to be 1.1 g, 80.35%. *M.p.* 478–479 K.

### Refinement

All hydrogen atoms were located from the difference Fourier map and refined freely.

### Crystal data

**Table:**

C_9_H_10_ClN_3_O	*F*(000) = 880
*M**_r_* = 211.65	*D*_x_ = 1.414 Mg m^−^^3^
Monoclinic, *C*2/*c*	Mo *K*α radiation, λ = 0.71073 Å
Hall symbol: -C 2yc	Cell parameters from 6351 reflections
*a* = 21.8191 (4) Å	θ = 3.1–32.1°
*b* = 7.0484 (1) Å	µ = 0.35 mm^−^^1^
*c* = 13.7249 (2) Å	*T* = 100 K
β = 109.633 (1)°	Plate, colourless
*V* = 1988.04 (6) Å^3^	0.41 × 0.20 × 0.03 mm
*Z* = 8	

### Data collection

**Table:**

Bruker SMART APEXII CCD area-detector diffractometer	3539 independent reflections
Radiation source: fine-focus sealed tube	2912 reflections with *I* > 2σ(*I*)
graphite	*R*_int_ = 0.052
φ and ω scans	θ_max_ = 32.3°, θ_min_ = 3.1°
Absorption correction: multi-scan (*SADABS*; Bruker, 2005)	*h* = −32→32
*T*_min_ = 0.867, *T*_max_ = 0.991	*k* = −10→10
28636 measured reflections	*l* = −20→20

### Refinement

**Table:**

Refinement on *F*^2^	Primary atom site location: structure-invariant direct methods
Least-squares matrix: full	Secondary atom site location: difference Fourier map
*R*[*F*^2^ > 2σ(*F*^2^)] = 0.039	Hydrogen site location: inferred from neighbouring sites
*wR*(*F*^2^) = 0.101	H atoms treated by a mixture of independent and constrained refinement
*S* = 1.04	*w* = 1/[σ^2^(*F*_o_^2^) + (0.049*P*)^2^ + 1.2937*P*] where *P* = (*F*_o_^2^ + 2*F*_c_^2^)/3
3539 reflections	(Δ/σ)_max_ = 0.001
167 parameters	Δρ_max_ = 0.45 e Å^−^^3^
0 restraints	Δρ_min_ = −0.27 e Å^−^^3^

### Special details

**Table:**

Experimental. The crystal was placed in the cold stream of an Oxford Cyrosystems Cobra open-flow nitrogen cryostat (Cosier & Glazer, 1986) operating at 100.0 (1) K.
Geometry. All e.s.d.'s (except the e.s.d. in the dihedral angle between two l.s. planes) are estimated using the full covariance matrix. The cell e.s.d.'s are taken into account individually in the estimation of e.s.d.'s in distances, angles and torsion angles; correlations between e.s.d.'s in cell parameters are only used when they are defined by crystal symmetry. An approximate (isotropic) treatment of cell e.s.d.'s is used for estimating e.s.d.'s involving l.s. planes.
Refinement. Refinement of *F*^2^ against ALL reflections. The weighted *R*-factor *wR* and goodness of fit *S* are based on *F*^2^, conventional *R*-factors *R* are based on *F*, with *F* set to zero for negative *F*^2^. The threshold expression of *F*^2^ > σ(*F*^2^) is used only for calculating *R*-factors(gt) *etc*. and is not relevant to the choice of reflections for refinement. *R*-factors based on *F*^2^ are statistically about twice as large as those based on *F*, and *R*- factors based on ALL data will be even larger.

### Fractional atomic coordinates and isotropic or equivalent isotropic displacement parameters (Å^2^)

**Table:**

	*x*	*y*	*z*	*U*_iso_*/*U*_eq_	
Cl1	0.023375 (16)	−0.37949 (4)	0.13113 (2)	0.02330 (9)	
O1	0.28038 (4)	0.80096 (12)	0.13075 (6)	0.01675 (17)	
N1	0.18652 (5)	0.38677 (13)	0.08164 (7)	0.01332 (18)	
N2	0.22047 (5)	0.53956 (13)	0.06342 (7)	0.01416 (18)	
N3	0.24395 (6)	0.61942 (14)	0.23627 (8)	0.0176 (2)	
C1	0.11370 (6)	−0.05633 (16)	−0.01844 (9)	0.0167 (2)	
C2	0.08397 (6)	−0.21004 (16)	0.01146 (10)	0.0178 (2)	
C3	0.06045 (6)	−0.18847 (16)	0.09274 (9)	0.0172 (2)	
C4	0.06590 (6)	−0.01713 (17)	0.14505 (9)	0.0184 (2)	
C5	0.09612 (6)	0.13476 (16)	0.11541 (9)	0.0167 (2)	
C6	0.12052 (6)	0.11705 (15)	0.03359 (9)	0.0136 (2)	
C7	0.15469 (6)	0.27915 (15)	0.00516 (9)	0.0137 (2)	
C8	0.24965 (6)	0.65995 (15)	0.14448 (8)	0.0136 (2)	
C9	0.15172 (7)	0.30648 (18)	−0.10430 (9)	0.0189 (2)	
H1	0.1299 (8)	−0.071 (2)	−0.0751 (13)	0.022 (4)*	
H2	0.0807 (8)	−0.332 (3)	−0.0235 (13)	0.029 (4)*	
H4	0.0472 (8)	−0.007 (2)	0.2047 (13)	0.026 (4)*	
H5	0.0998 (8)	0.254 (2)	0.1502 (13)	0.024 (4)*	
H9A	0.1211 (11)	0.237 (3)	−0.1478 (18)	0.053 (6)*	
H9B	0.1901 (12)	0.271 (3)	−0.1107 (18)	0.064 (7)*	
H9C	0.1416 (9)	0.433 (3)	−0.1261 (15)	0.036 (5)*	
H1N2	0.2197 (8)	0.579 (3)	0.0019 (14)	0.028 (4)*	
H1N3	0.2668 (8)	0.684 (2)	0.2863 (13)	0.022 (4)*	
H2N3	0.2271 (8)	0.522 (2)	0.2487 (12)	0.020 (4)*	

### Atomic displacement parameters (Å^2^)

**Table:**

	*U*^11^	*U*^22^	*U*^33^	*U*^12^	*U*^13^	*U*^23^
Cl1	0.02608 (17)	0.01993 (15)	0.02367 (16)	−0.00699 (11)	0.00806 (12)	0.00422 (10)
O1	0.0227 (4)	0.0154 (4)	0.0128 (4)	−0.0054 (3)	0.0067 (3)	−0.0010 (3)
N1	0.0157 (4)	0.0121 (4)	0.0120 (4)	−0.0013 (3)	0.0044 (3)	0.0007 (3)
N2	0.0202 (5)	0.0127 (4)	0.0100 (4)	−0.0042 (3)	0.0056 (4)	−0.0007 (3)
N3	0.0272 (6)	0.0163 (4)	0.0102 (4)	−0.0065 (4)	0.0076 (4)	−0.0015 (3)
C1	0.0176 (5)	0.0165 (5)	0.0163 (5)	−0.0024 (4)	0.0064 (4)	−0.0024 (4)
C2	0.0183 (6)	0.0141 (5)	0.0204 (6)	−0.0024 (4)	0.0055 (4)	−0.0019 (4)
C3	0.0165 (5)	0.0148 (5)	0.0188 (5)	−0.0024 (4)	0.0039 (4)	0.0037 (4)
C4	0.0200 (6)	0.0194 (5)	0.0166 (5)	−0.0019 (4)	0.0074 (4)	0.0013 (4)
C5	0.0200 (6)	0.0151 (5)	0.0158 (5)	−0.0013 (4)	0.0073 (4)	−0.0010 (4)
C6	0.0138 (5)	0.0142 (5)	0.0121 (5)	−0.0004 (4)	0.0034 (4)	0.0013 (4)
C7	0.0158 (5)	0.0127 (4)	0.0121 (5)	−0.0006 (4)	0.0040 (4)	0.0003 (4)
C8	0.0164 (5)	0.0129 (4)	0.0109 (5)	−0.0002 (4)	0.0040 (4)	−0.0005 (4)
C9	0.0266 (7)	0.0185 (5)	0.0124 (5)	−0.0052 (5)	0.0077 (5)	−0.0008 (4)

### Geometric parameters (Å, °)

**Table:**

Cl1—C3	1.7410 (12)	C2—C3	1.3842 (18)
O1—C8	1.2481 (13)	C2—H2	0.977 (18)
N1—C7	1.2922 (14)	C3—C4	1.3895 (17)
N1—N2	1.3768 (13)	C4—C5	1.3882 (16)
N2—C8	1.3737 (14)	C4—H4	1.033 (17)
N2—H1N2	0.883 (18)	C5—C6	1.4006 (16)
N3—C8	1.3378 (14)	C5—H5	0.958 (17)
N3—H1N3	0.835 (18)	C6—C7	1.4863 (15)
N3—H2N3	0.826 (17)	C7—C9	1.4943 (16)
C1—C2	1.3935 (16)	C9—H9A	0.88 (2)
C1—C6	1.3979 (15)	C9—H9B	0.91 (2)
C1—H1	0.963 (16)	C9—H9C	0.94 (2)
			
C7—N1—N2	119.12 (9)	C4—C5—C6	120.81 (11)
C8—N2—N1	117.86 (9)	C4—C5—H5	120.0 (10)
C8—N2—H1N2	115.9 (12)	C6—C5—H5	119.1 (10)
N1—N2—H1N2	125.4 (12)	C1—C6—C5	118.92 (10)
C8—N3—H1N3	116.1 (12)	C1—C6—C7	120.97 (10)
C8—N3—H2N3	124.2 (11)	C5—C6—C7	120.08 (10)
H1N3—N3—H2N3	118.0 (16)	N1—C7—C6	114.71 (10)
C2—C1—C6	120.63 (11)	N1—C7—C9	124.82 (10)
C2—C1—H1	119.1 (10)	C6—C7—C9	120.46 (10)
C6—C1—H1	120.3 (10)	O1—C8—N3	122.47 (10)
C3—C2—C1	119.16 (11)	O1—C8—N2	119.73 (10)
C3—C2—H2	120.4 (10)	N3—C8—N2	117.80 (10)
C1—C2—H2	120.4 (10)	C7—C9—H9A	112.1 (14)
C2—C3—C4	121.43 (11)	C7—C9—H9B	109.4 (15)
C2—C3—Cl1	119.65 (9)	H9A—C9—H9B	107 (2)
C4—C3—Cl1	118.92 (9)	C7—C9—H9C	111.6 (11)
C5—C4—C3	119.04 (11)	H9A—C9—H9C	105.6 (19)
C5—C4—H4	122.2 (10)	H9B—C9—H9C	110.9 (19)
C3—C4—H4	118.7 (10)		
			
C7—N1—N2—C8	175.03 (10)	C4—C5—C6—C7	−177.97 (11)
C6—C1—C2—C3	0.79 (18)	N2—N1—C7—C6	179.22 (9)
C1—C2—C3—C4	−0.05 (18)	N2—N1—C7—C9	0.30 (17)
C1—C2—C3—Cl1	−179.84 (9)	C1—C6—C7—N1	−146.43 (11)
C2—C3—C4—C5	−0.55 (19)	C5—C6—C7—N1	31.82 (15)
Cl1—C3—C4—C5	179.24 (9)	C1—C6—C7—C9	32.55 (16)
C3—C4—C5—C6	0.41 (18)	C5—C6—C7—C9	−149.20 (12)
C2—C1—C6—C5	−0.92 (18)	N1—N2—C8—O1	−179.37 (10)
C2—C1—C6—C7	177.35 (11)	N1—N2—C8—N3	0.92 (16)
C4—C5—C6—C1	0.32 (17)		

### Hydrogen-bond geometry (Å, °)

**Table:**

*D*—H···*A*	*D*—H	H···*A*	*D*···*A*	*D*—H···*A*
N2—H1N2···O1^i^	0.884 (19)	2.007 (19)	2.8866 (12)	173.3 (19)
N3—H1N3···N1^ii^	0.835 (18)	2.264 (18)	3.0904 (14)	170.5 (16)
N3—H2N3···O1^iii^	0.826 (17)	2.316 (17)	3.0499 (13)	148.4 (15)
C9—H9C···O1^i^	0.94 (2)	2.55 (2)	3.2162 (16)	128.1 (16)

Symmetry codes: (i) −*x*+1/2, −*y*+3/2, −*z*; (ii) −*x*+1/2, *y*+1/2, −*z*+1/2; (iii) −*x*+1/2, *y*−1/2, −*z*+1/2.
